# Simultaneous zero echo time fMRI of rat brain and spinal cord

**DOI:** 10.1002/mrm.30633

**Published:** 2025-07-17

**Authors:** Hanne Laakso, Lin Wu, Sara Ponticorvo, Raimo A. Salo, Jaakko Paasonen, Ekaterina Paasonen, Mikko Kettunen, Russell L. Lagore, Lance DeLabarre, Ethan Polcyn, Gregor Adriany, Javier Istúriz, Dee M. Koski, Djaudat Idiyatullin, Olli Gröhn, Silvia Mangia, Shalom Michaeli

**Affiliations:** ^1^ A. I. Virtanen Institute for Molecular Sciences University of Eastern Finland Kuopio Finland; ^2^ Center for Magnetic Resonance Research, Deptartment of Radiology University of Minnesota Minneapolis Minnesota USA; ^3^ Kuopio University Hospital, Neurocenter Kuopio Finland; ^4^ Neos Biotec Pamplona Spain

**Keywords:** brain fMRI, dual‐FOV, MB‐SWIFT, simultaneous acquisition, spinal cord fMRI, zero‐TE

## Abstract

**Purpose:**

Functional assessments of the central nervous system (CNS) are essential for many areas of research. fMRI typically targets either the brain or the spinal cord, but usually not both, because of the obstacles associated with simultaneous image acquisitions from distant fields of view (FOVs) with conventional MRI. In this work, we establish a novel MRI approach that enables artifact‐free, quiet, simultaneous fMRI of both brain and spinal cord, avoiding the need for dynamic shimming procedures.

**Methods:**

We used zero TE Multi‐Band‐SWeep Imaging with Fourier Transformation (MB‐SWIFT) technique at 9.4 T in a simultaneous dual‐FOV configuration and two separate RF transmit‐receive surface coils. The first coil covered the rat brain, while the second was positioned approximately at the T13‐L1 level of the rat's spinal cord with copper shielding to minimize the coupling between the RF coils. Eight Sprague–Dawley rats were used for hindlimb stimulation fMRI studies.

**Results:**

Robust and specific activations were detected in both the brain and spinal cord during hindlimb stimulation at individual and group levels. The results established the feasibility of the novel approach for simultaneous functional assessment of the lumbar spinal cord and brain in rats.

**Conclusion:**

This study demonstrated the feasibility of a novel dual‐FOV fMRI approach based on zero‐TE MB‐SWIFT and set the stage for translation to humans. The methodology enables comprehensive functional CNS evaluations of great value in different conditions such as pain, spinal cord injury, neurodegenerative diseases, and aging.

## 1 INTRODUCTION

fMRI has revolutionized the field of neuroscience by enabling the non‐invasive visualization of hemodynamic and metabolic processes linked to neuronal activity in vivo. It plays a crucial role in mapping brain function and network connectivity in both humans and animals. With the rapid development of advanced MRI techniques, it has become feasible to evaluate microstructure and function with high spatial sensitivity and specificity. However, all currently available pulse sequences used in functional or structural MRI research have predominantly focused on one organ, such as the brain or the spinal cord. In recent years, there has been increasing interest in expanding the capabilities of fMRI to encompass the spinal cord.^1^, ^2^ Despite the growing interest and advancements in acquisition and processing strategies,^3^ spinal cord fMRI remains underdeveloped compared to brain fMRI. Susceptibility artifacts arising from tissue characteristics, motion of the spinal cord and cerebrospinal fluid induced by cardiac and respiratory cycles, and the relatively small cross‐sectional dimension of the spinal cord compromise the quality of the images.^3^, ^4^

Only a limited number of fMRI studies combining brain and spinal cord fMRI have been reported,^5^, ^6^, ^7^, ^8^, ^9^, ^10^, ^11^, ^12^ mostly including only midbrain regions and the cervical spinal cord. However, neither more extensive coverage of the brain and spinal cord nor simultaneous fMRI has been reported so far. In fact, despite the potential impact of a comprehensive fMRI approach for studying the central nervous system (CNS), achieving such a goal poses considerable challenges. These are largely attributed to technical obstacles with conventional MRI techniques, particularly the necessity for high magnetic field homogeneity across a large field of view (FOV) or several FOVs capable of encompassing both the brain and spinal cord simultaneously. To overcome this issue, per‐slice dynamic shimming approaches have been proposed.^4^, ^13^ However, they present drawbacks, including a notable extension of scanning time, limitations imposed by the settling‐time of eddy currents, and applications primarily restricted to covering the cervical, but not lower regions of the spinal cord. Furthermore, within a single repetition time, only sequential rather than interleaved FOV acquisitions of the brain and spinal cord have been achievable, hindering the realization of simultaneous acquisitions optimal for comprehensive connectivity analyses of the CNS.

In this work, we used the zero‐TE MRI technique called Multi‐Band‐SWeep Imaging with Fourier Transformation (MB‐SWIFT)^14^, ^15^, which was modified to acquire signals from two FOVs (dual‐FOV) simultaneously. MB‐SWIFT, as other zero‐TE sequences, is minimally affected by field inhomogeneities, therefore, inherently addressing most of the above‐mentioned issues because of high bandwidth. We previously demonstrated the feasibility and numerous technical benefits of MB‐SWIFT for studying the function of the rat brain^16^, ^17^, ^18^, ^19^ and spinal cord^20^ separately. We have also shown that the functional contrast in MB‐SWIFT fMRI originates from the inflow of unsaturated blood,^17^, ^21^ differentiating it from the traditional BOLD contrast.^22^ Here, we used this novel MRI approach for artifact‐free, quiet fMRI simultaneously from the brain and lumbar spinal cord during hindlimb stimulation, avoiding the need for dynamic shimming approaches.^13^

## 2 METHODS

### 2.1 MB‐SWIFT pulse sequence with dual‐FOV capabilities

**FIGURE 1:**
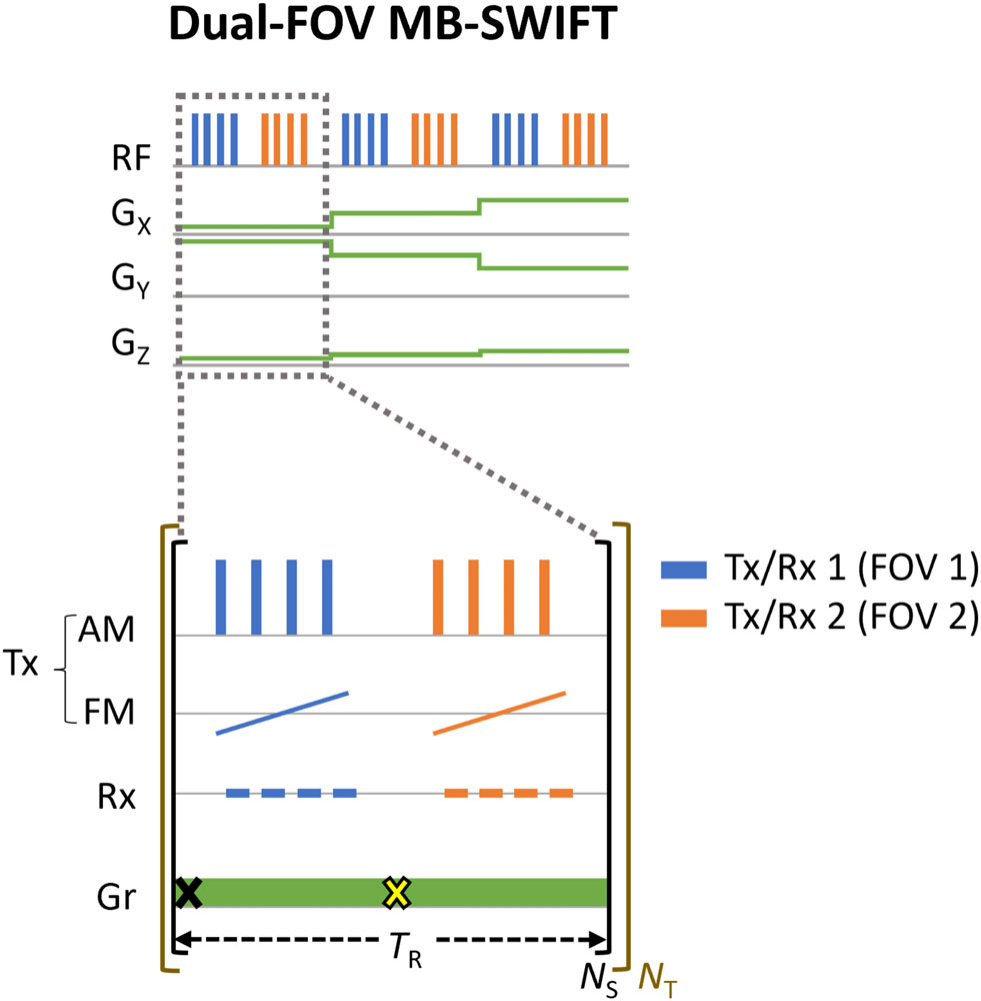
Schematic view of the dual‐FOV Multi‐Band‐SWeep Imaging with Fourier Transformation (MB‐SWIFT) sequence for simultaneous acquisition of the two FOVs. *T*_R_, repetition time for the two spokes (1.94 ms in our setup); *N*_S_, number of spokes to acquire one image volume (= 1547); *N*_T_, number of time points (image series) (= 248), AM, amplitude modulation function; FM, frequency modulation function, Tx1 and Tx2 transmitting channels, Rx1 and Rx2 receiving channels. The diagram represents MB‐SWIFT pulse sequence with 4 gaps following 4 excitation pulse elements; Gr, field gradient (green bar) keeps the same orientation for both spokes and changing its orientation during about 0.3 ms once per each *T*_R_, as indicated by the black crossed lines. Change in the gradient amplitude between the spokes, indicated by the yellow crossed lines, is used in the case when two FOVs of different sizes are used. In our setup volume‐TR was 3 s.

In Figure 1, the schematic representation of the radial pulse sequence modified from the original MB‐SWIFT technique for dual‐FOV acquisitions is presented. The pulse sequence allows for virtually simultaneous (up to ˜1 ms repetition time per spoke) acquisitions of two independent FOVs. As in the original MB‐SWIFT method, the orientation of the gradient changes incrementally from spoke to spoke. During a given gradient orientation, sequential excitations and readouts are performed for the independent FOVs irradiated by two different RF coils. Acquisition is then repeated *N*_S_ times, and therefore, the dual‐FOV volume is acquired with an overall *N*_S_ *T*_R_ time resolution, where *T*_R_ is the repetition time between the excitation of the same FOV (i.e., *T*_R_ = 2 × spoke repetition time). The offset frequencies of the transmitters change according to the location of FOV. The amplitude of the readout gradient can be separately adjusted to allow the different sized FOVs. The number of pulses (number of gaps in the gapped pulse) can be increased to gain SNR and power efficiency or decreased for faster imaging. The MB‐SWIFT with the dual‐FOV capabilities is freely available for non‐commercial use at https://license.umn.edu/product/swift‐software‐for‐bruker‐mri‐systems#! for Bruker and at https://license.umn.edu/product/vmnrj‐swift‐software‐for‐agilent‐varian‐systems#! for Agilent platforms, respectively.

### 2.2 Hardware configuration for dual‐FOV MB‐SWIFT fMRI

**FIGURE 2:**
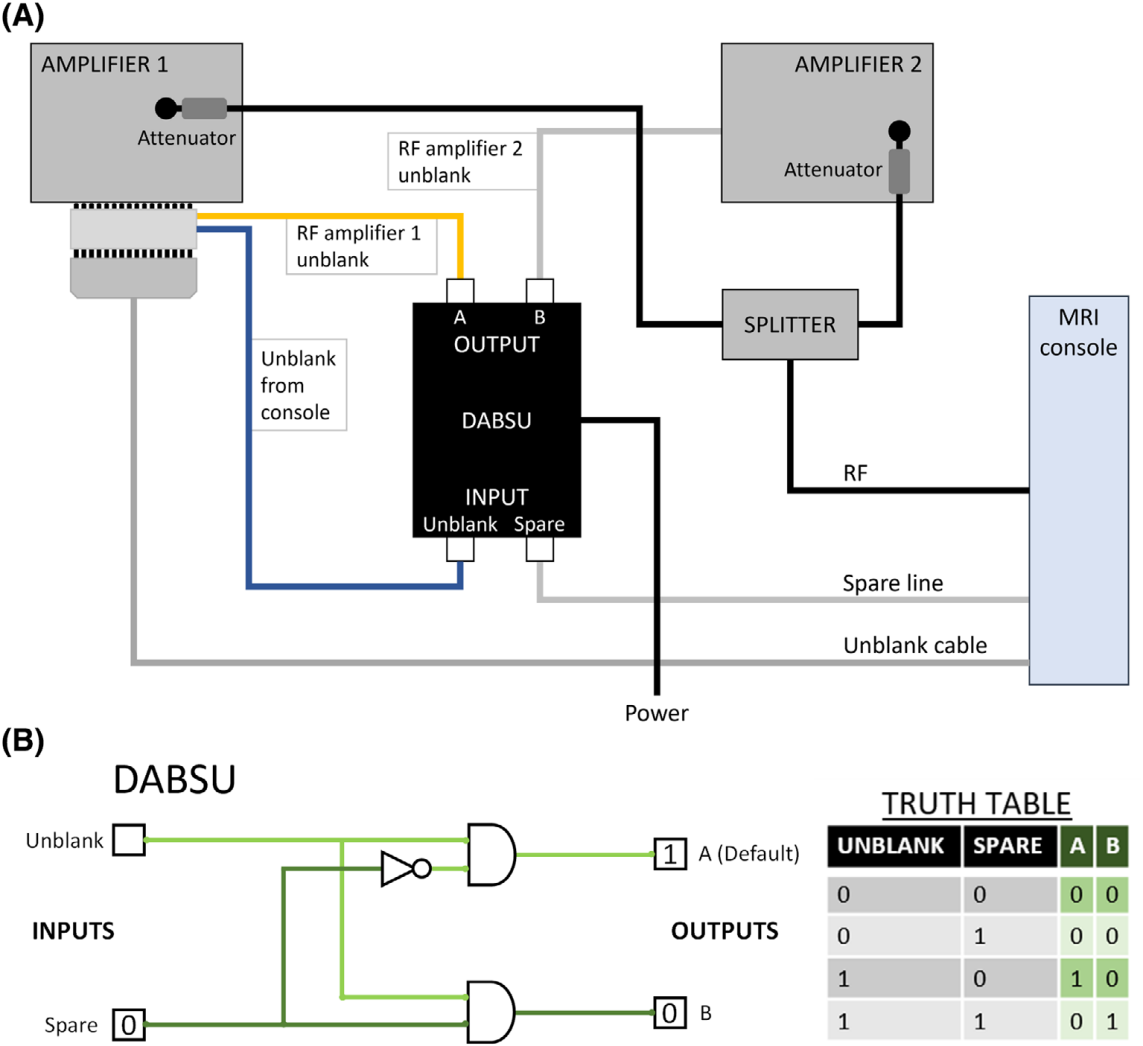
Schematic overview of the setup for dual‐FOV. (A) Connections from the console to Dual Amplifier Blanking Selection Unit (DABSU) controlling the unblanking of the two individual amplifiers. (B) Schematic diagram of DABSU. The input “Unblank” signal is distributed to output channel “A” or “B” depending on the signal level on input called “Spare”. The “Unblank” and “Spare” signals are supplied from the scanner's console. The “A” and “B” outputs are connected to the “Unblank” inputs of two amplifiers.

Figure 2A shows the setup and connections for the dual‐FOV imaging at our scanner. Two RF amplifiers are required to be alternated accordingly to transmit the signal to a given coil, and for this, a Dual Amplifier Blanking Selection Unit (DABSU) was developed because our system does not support parallel transmission (pTx). The DABSU enables alternately unblanking two independent RF amplifiers for excitation of two distant FOVs (Figure 2). In the instruments where pTx is available, transition of the signal to two separate coils could be achieved without DABSU.

The DABSU unit is a simple 5‐volt digital logic device based on 7400‐series (Texas Instruments) integrated circuits, which intercepts the transistor‐transistor logic (TTL) unblank signal from the console and also accepts an additional (spare) TTL signal from the console and uses these inputs to generate two alternating unblank signals for the two independent RF amplifiers (Figure 2B). This enables alternated independent excitation of two FOVs. The truth table presented in Figure 2 shows the two outputs (A and B) produced from the two inputs (spare and unblank). Unblank goes high during the transmit pulse, whereas the spare is switched either low to unblank the RF amplifier connected to output A or high to unblank the RF amplifier connected to output B.

**FIGURE 3:**
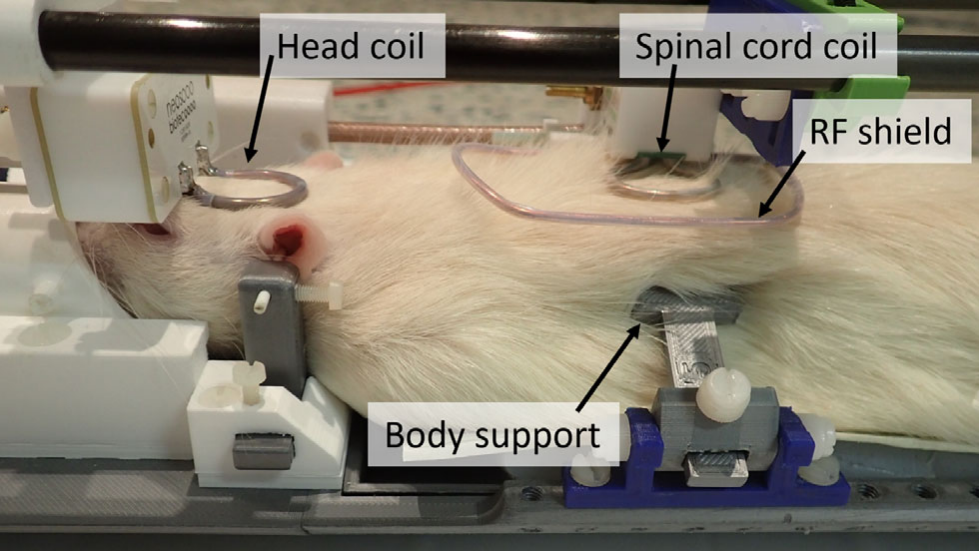
Setup for simultaneous brain and spinal cord fMRI. One oval shaped loop coil (inner axes 14 × 16 mm) was placed on the head and another oval shaped loop coil (inner axes 14 × 18 mm) was placed on the spinal cord of a rat at the vertebral level T13‐L1. An adjustable RF shield loop (inner axes 34 × 62 mm, copper wire) was placed around the spinal cord coil to minimize coupling between the two coils. The body of the rat was supported with bars stabilizing the spine from the sides to reduce the motion from breathing.

Two individual transmit‐receive RF loop coils were used at 9.4 T equipped with the Agilent DirectDRIVE console. The first oval shaped coil (inner axes 14 × 16 mm, Neos Biotec) covered the rat brain, while the second coil (inner axes 14 × 18 mm, Neos Biotec) was positioned approximately at the T13‐L1 vertebral level of the rat spine with an adjustable RF shield loop (inner axes 34 × 62 mm, copper wire) placed around it to minimize coupling between the coils (Figure 3). This shielding loop consists of a closed loop of copper wire, only interrupted by a DC isolation capacitor, to prevent the flow of gradient‐induced eddy currents, while allowing a free circulation of RF currents. The position of this RF shield loop is adjusted to an optimum spot where the magnetic flux directly coupled between head and spine coils cancels out with the flux indirectly coupled between them (through the circulation of an induced current in the shielding loop). Using the RF shield, we were able to achieve average decoupling of approximately −40 dB as measured on a network analyzer (NanoVNA‐F V2, SYSJOINT Information Technology).

For dual‐FOV acquisitions we performed first order shimming based on the linewidth of spectra from signals of each FOV. Mostly Z‐shimming gradient was adjusted to reach the same central offset frequency for both channels. This was achieved by performing global shimming on a large slab covering both brain and spinal cord, referred here to as average shim.

### 2.3 Animal experiments

Eight Sprague–Dawley rats (weight 305 g ± 49 g [mean ± SD], 3 females and 5 males) were used for the studies. Food and water were available ad libitum. All animal procedures were approved by the Finnish Animal Experiment Board and conducted in accordance with the European Commission Directive 2010/63/EU guidelines.

The rats were initially anesthetized with isoflurane (5% induction, 2% during setup, carrier gas O_2_/N_2_ = 30/70%) and then a bolus of 0.015 mg/kg medetomidine was given subcutaneously. The animal was placed on the holder and the head was fixed with toothbar and earbars, while the body of the rat was supported with bars stabilizing the spine from the sides to reduce breathing‐induced motion (Figure 3). A warm water circulating heating pad was placed under the animal to keep the animal's temperature close to 37°C. An infusion line with a needle was inserted subcutaneously into the back of the rat and after 15 min from the medetomidine bolus, an infusion of medetomidine, 0.03 mg/kg/h, was started and the isoflurane was reduced to approximately 0.5%. Two needle electrodes were placed under the skin on the sides of the right heel pointing proximally to the leg of the animal, to stimulate the fascicles of the sciatic nerve. Before scanning, the stimulation was tested to observe slight movement of the paw induced by the electrical stimulation. Respiration and temperature of the animal were monitored during the scans using a pneumatic pillow under the animal and a rectal temperature probe (Model 1025, Small Animal Instruments), respectively. After the experiment, atipamezole, 0.5 mg/mL, 0.5 mg/kg was given to reverse the sedative effect of medetomidine.

### 2.4 fMRI

MB‐SWIFT technique modified for simultaneous dual‐FOV acquisitions was used for fMRI acquisitions with 3 s temporal resolution and 1547 spokes per image volume, acquired in simultaneous fashion (interleaved spokes) with spiral trajectory. The other parameters were *T*_R_ = 1.94 ms (single spoke acquisition time was 0.97 ms), FOV bandwidth (BW) =192 kHz, acquisition BW was BW*os = 384 kHz, where os is oversampling = 2, number of samples per spoke 32*os, baseband sweep range (range of frequency modulation function) BW/32 = 6 kHz, excitation/acquisition BW = 192/384 kHz, FOV = 40 × 40 × 40 mm^3^, flip angle = 6°, matrix = 64^3^, and 625 μm isotropic resolution for both brain and spinal cord. The acquisition of signal (readout) used the gaps between four short excitation pulse elements: pulse element width = 2.6 μs, post excitation delay = 2.5 μs, pre‐excitation delay = 0.1 μs, readout time of 32 points in each gap 167.5 μs and in total 670 μs, and gradient changing time 292.5 μs per spoke. In total 248 volumes per FOV were collected during the fMRI scans, resulting in 12 min 24 s acquisition time.

An anatomical high‐resolution image was acquired using magnetization transfer (MT) weighted MB‐SWIFT with similar parameters, but with 4000 spokes per spiral, 16 spirals, 8° flip angle, and 256^3^ matrix, 156 μm isotropic resolution. For MT, a sinc pulse placed at 1500 Hz off‐resonance was used. Pulse duration of the sinc pulse was 15 ms, given every 32 spokes at the amplitude γB_1_ = 167 Hz. The total acquisition time for the anatomical scan was 8 min for the two FOVs.

In four rats, we also conducted comparative scans using single‐FOV MB‐SWIFT acquisitions with time resolution of 1.5 s and 496 volumes in the brain and spinal cord separately. For those acquisitions, the flip angle was reduced to 4° to compensate for the effect of shorter repetition time to achieve the same Ernst condition.

Furthermore, we performed an additional experiment in one animal with dual‐FOV and single‐FOV MB‐SWIFT and comparative spin echo (SE)‐EPI acquisitions with parameters TR = 3 s, TE = 35 ms, FOV = 40 × 40 mm^2^, 18 slices 1 mm thick. Two shimming strategies were used for EPI: first, averaged for both coils as in dual‐FOV, and second, targeted for the imaged FOV.

The stimulation paradigm started with 60 s of rest followed by a 24‐s block of stimulation and 90 s of rest repeated 6 times. The stimulation block consisted of 600 μs symmetric charge balanced pulses at 9 Hz frequency and 2 mA current. We used STG4008 stimulus generator (Multi Channel Systems [MCS]) in the current mode to deliver the stimulation paradigm, which was synchronized to start with the first volume of the fMRI acquisition by a TTL trigger pulse from the scanner.

### 2.5 Reconstruction and preprocessing

The MB‐SWIFT data were reconstructed as in Dvořáková et al.^23^ using RF‐pulse deconvolution, gridding andfast iterative shrinkage‐thresholding algorithm^24^ with 13 iterations. All MRI data were processed and analyzed with in‐house made scripts, using Snakemake^25^ (https://snakemake.github.io/), Python (version 3.10, https://www.python.org/downloads/), advanced normalization tools^26^ (ANTs) (https://stnava.github.io/ANTs/), FSL (version 6.0, https://fsl.fmrib.ox.ac.uk/fsl/fslwiki/), and FSL FEAT.^27^

Motion correction was performed by aligning the time series images to the first volume using ANTs.^28^, ^29^ An independent component analysis (ICA) (https://fsl.fmrib.ox.ac.uk/fsl/fslwiki/MELODIC) based motion regression was conducted to further improve the data quality by removing components representing motion, similarly as in Dvořáková et al.^30^ The motion components were automatically selected from the obtained 15 components and regressed out. Motion in the image time series was estimated by the movement of the signal center of mass in three dimensions. To define the component as motion, two criteria were set: (1) when the correlation of the time series of the component with one of the motion parameters exceeded 0.75, and (2) when more than 70% of the spatial map voxels were located on the edge regions of the brain or outside, on top of the spinal cord.

Co‐registration of the anatomical images for group level analysis was done in two steps using ANTs. First, separately for the brain and spinal cord images, five landmarks were selected and labeled from each of the images to make an initial rigid co‐registration to reference brain and spinal cord selected from the data. Second, a nonlinear symmetric normalization (SyN) registration was applied to refine the rigid registration. Subsequently, the registration transformations were applied to the functional data.

For the separate data set collected for comparison with EPI, motion correction was performed with ANTs similarly as with the other data, but no ICA based motion regression was conducted for either EPI or MB‐SWIFT. The EPI data was slice time corrected and motion correction was done using the same protocol with ANTs as with the MB‐SWIFT data.

For the subject‐wise activation maps, the time series were high‐pass filtered (0.01 Hz) and the autocorrelation was removed. The maps were calculated with FSL FEAT using a general linear model (GLM). In particular, the stimulation periods were modeled as boxcar functions and convolved with a γ‐variate impulse response function (IRF) assuming delay of the response 2.8 s and variation 1.4 s resembling the previously reported IRFs for rodents.^21^ To enable fair comparison between single‐FOV and dual‐FOV acquisitions, from single‐FOV MB‐SWIFT data every other volume was taken into account for calculating the activation map with the same time resolution of the dual‐FOV MB‐SWIFT dataset. The group‐level activation maps were estimated using the FSL nonparametric permutation tool Randomise^31^ with the threshold‐free cluster enhancement (tfce) test statistic, with *p*‐values corrected for family‐wise error (FWE) rate and values of *p* < 0.05 considered as significant.

## 3 RESULTS

**FIGURE 4:**
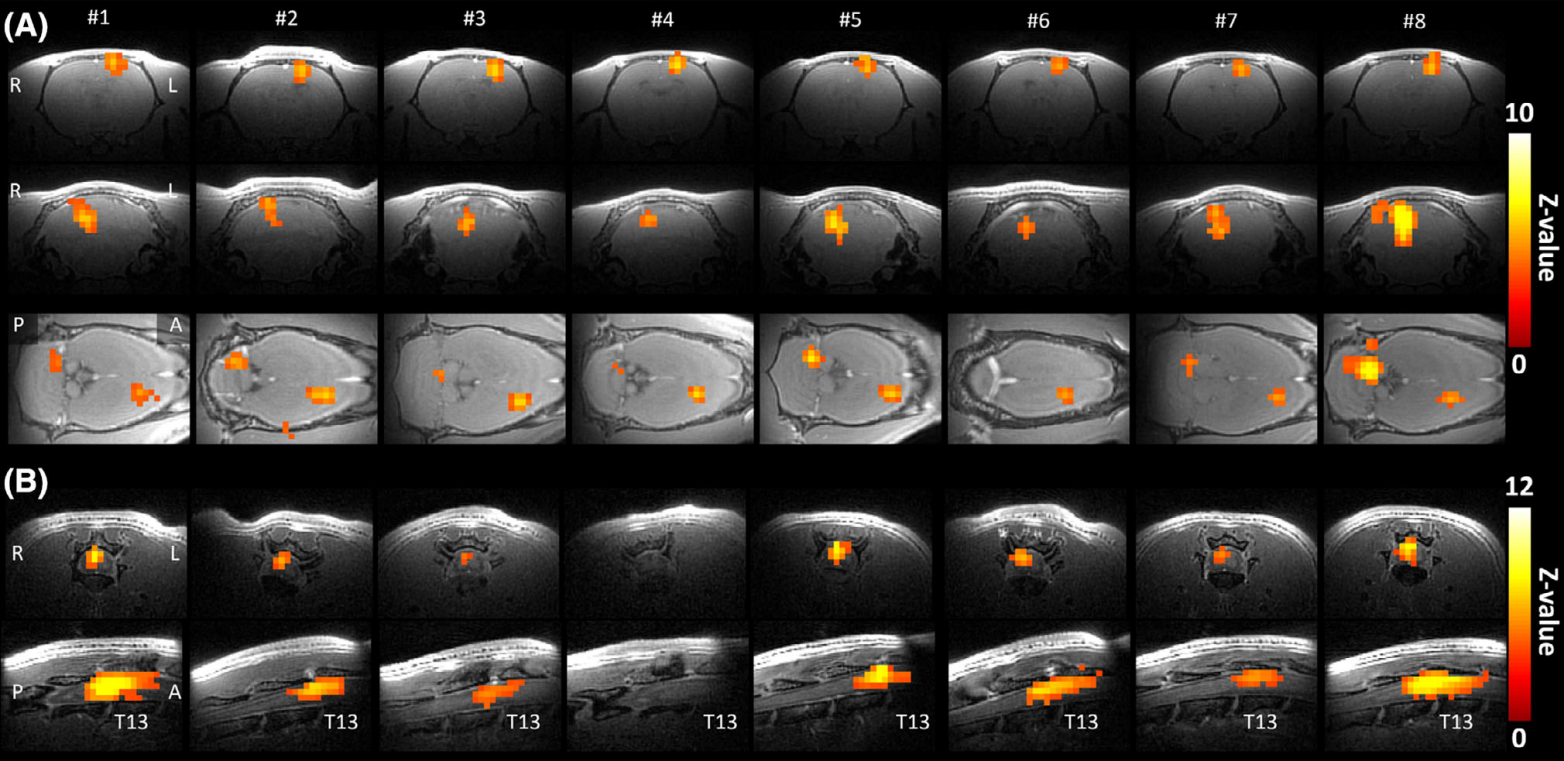
Dual‐FOV Multi‐Band‐SWeep Imaging with Fourier Transformation (MB‐SWIFT) fMRI activation maps from (A) brain at cortex and cerebellum and (B) spinal cord from individual animals during hindlimb stimulation. In the brain, the activations are located in the contralateral somatosensory and motor cortex and ipsilateral cerebellum. In the spinal cord, the activation is ipsilateral, mostly on the dorsal side at T13‐L1 vertebral level (L4 spinal). Threshold‐free cluster enhancement (tfce) z > 3.1 thresholded. R, right; L, left; P, posterior; A, anterior; T13, the level of T13 vertebra.

**FIGURE 5:**
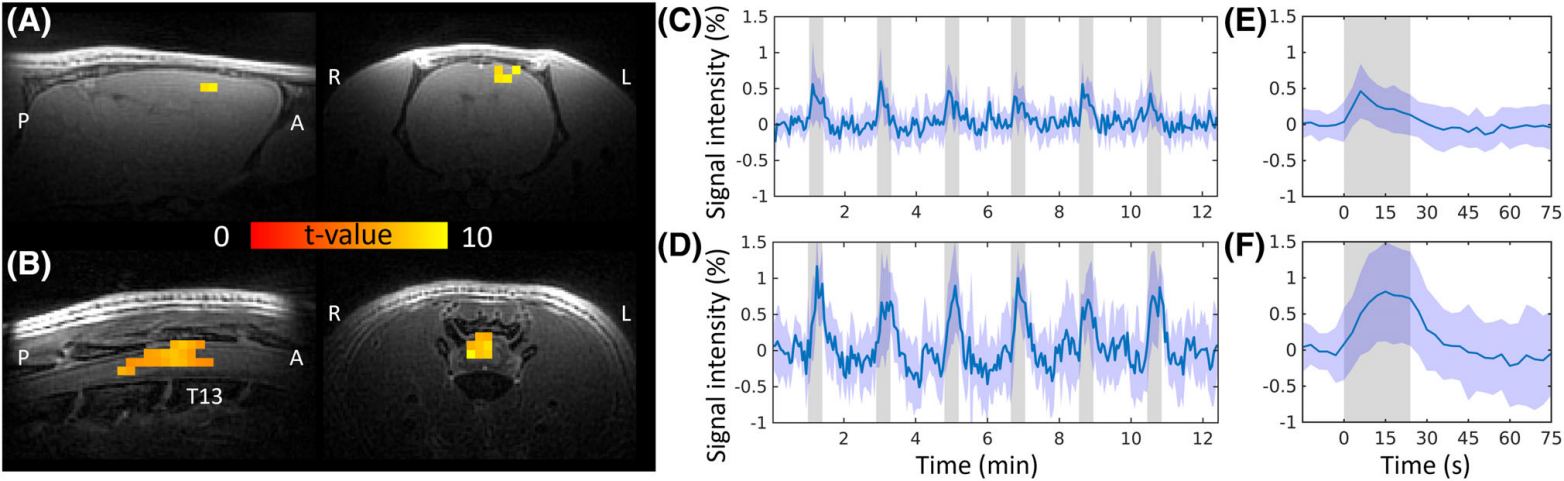
Group level analysis of dual‐FOV Multi‐Band‐SWeep Imaging with Fourier Transformation (MB‐SWIFT) fMRI during hindlimb stimulation. The activation at group level is localized in the left somatosensory cortex (A) and right dorsal spinal cord at L4 spinal level (B). Threshold‐free cluster enhancement (tfce) *p* < 0.05, family‐wise error (FWE) corrected. The time courses (C,D) show the mean time courses from the activation clusters shown on the same row, and the shaded area represents the SD across subjects. The mean activation across subjects and scans for a single stimulation block is shown for brain (E) and spinal cord (F). The gray bars depict the stimulation timings. R, right; L, left, P, posterior; A, anterior; T13, the level of T13 vertebra.

**FIGURE 6:**
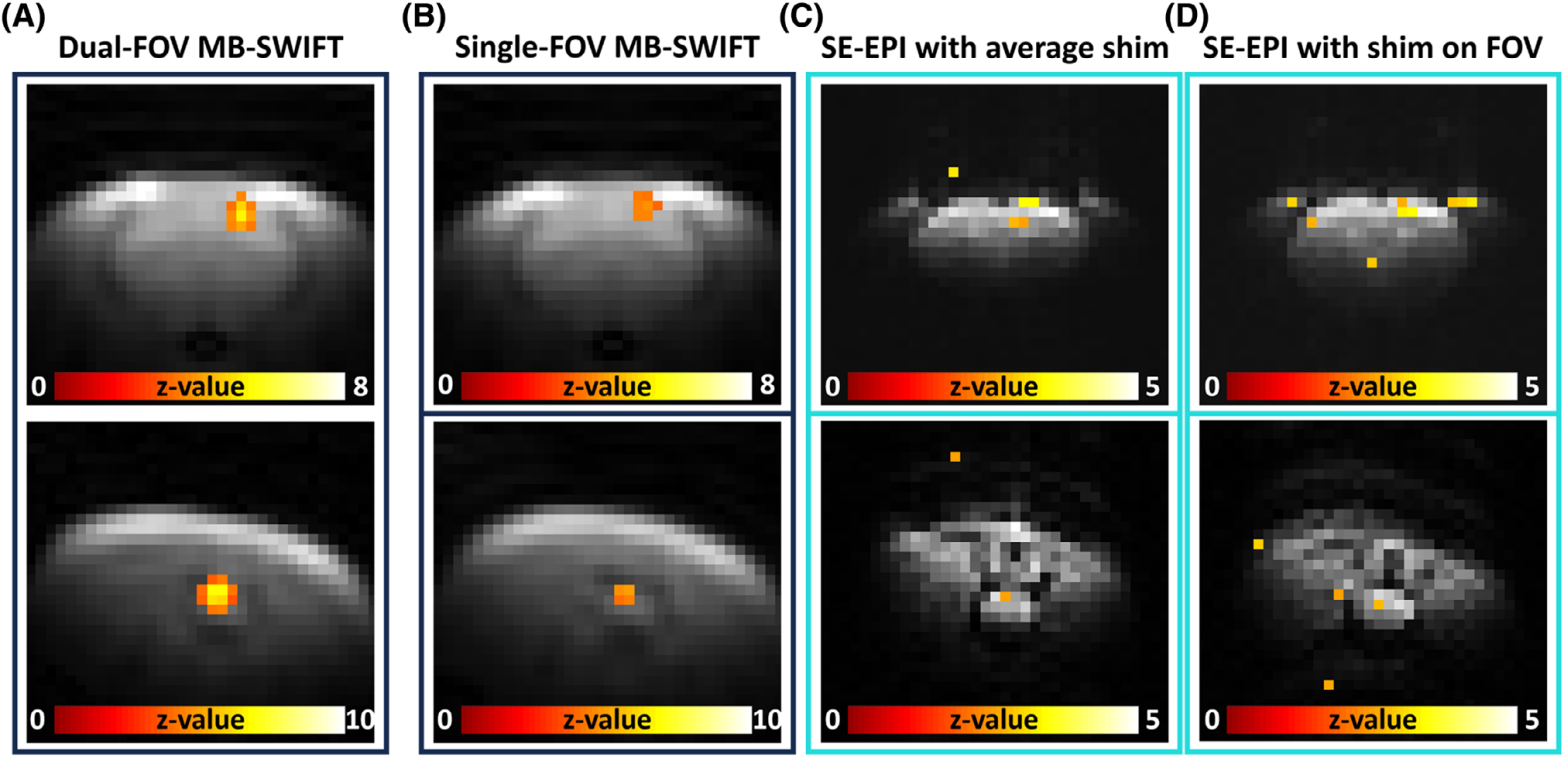
Comparison between dual‐FOV and single‐FOV Multi‐Band‐SWeep Imaging with Fourier Transformation (MB‐SWIFT) and spin echo (SE)‐EPI fMRI during hindlimb stimulation. fMRI activation with: (A) simultaneous MB‐SWIFT acquisition from two FOVs with average shim; (B) with separate single‐FOV MB‐SWIFT acquisitions with average shim; (C) separate SE‐EPI acquisitions with average shim; (D) separate SE‐EPI acquisitions with shimming performed on the target FOV. Threshold‐free cluster enhancement (tfce) z > 3.1 thresholded. The fMRI activation maps are overlaid on the original functional scans to show image quality.

The dual‐FOV MB‐SWIFT acquisitions using average shimming on both FOVs provided good image quality and stimulation‐based functional contrast in both FOVs. Robust and specific activations were detected in both the brain and spinal cord during hindlimb stimulation in seven of eight rats (Figure 4). Specifically, in the brain, the responses localized to the contralateral somatosensory and motor cortex and ipsilateral cerebellum (Figure 4A). In the spinal cord, the activated area was at T13‐L1 vertebral level (L4 spinal) ipsilateral to the stimulation and mostly in the dorsal horn (Figure 4B). The cortical cluster size was 18 to 39 voxels across animals with cluster‐averaged z‐value between 4.3 and 5.8 (Table S1). In the spinal cord, the cluster size was 42 to 166 voxels across animals with cluster‐averaged z‐values 4.3 to 6.8 (Table S1). At group level, we detected clear responses in the spinal cord at L4 spinal level in the ipsilateral dorsal horn and in the brain in the contralateral somatosensory and motor cortex (Figure 5A,B). The time courses obtained from the areas depicted by the group level activation also show clear response to the stimulation both in the brain (˜0.5% signal change) and in the spinal cord (˜1% signal change) (Figure 5C,D). The responses in brain and spinal cord show different time courses; the response peaks approximately 9 s after the stimulation start in brain and after approximately 18 s in the spinal cord (Figure 5E,F). Furthermore, the return to baseline appears to take longer in spinal cord (˜45 s) compared to brain (˜33 s). Individual animal responses show similar differences between the response timings (Figures S1, S2), and the peak response times were significantly different (*p* < 0.001, paired *t* test) between the brain and the spinal cord. Dual‐FOV MB‐SWIFT and single‐FOV MB‐SWIFT data showed similar activation patterns both in the brain and spinal cord (Figure 6A,B) and no significant differences were found between the activations (*p* > 0.05, FWE corrected, Randomise).

When compared to EPI scans with average shim, MB‐SWIFT images of the spinal cord were less distorted and enabled robust detection of activation in the spinal cord (Figure 6). Moreover, only a few statistically significant voxels not forming a clear cluster were detected in the spinal cord with EPI and single activated voxels were seen elsewhere, also outside the rat body, indicating lower quality of data. When shimming individually to the imaged FOVs, (i.e., brain or spinal cord) the EPI images were slightly less distorted than with average shim, but the activation patterns were still sparse as compared to MB‐SWIFT (Figure 6).

## 4 DISCUSSION

In this study, we demonstrated that MB‐SWIFT enables comprehensive stimulation‐based fMRI studies of the CNS by acquiring signals virtually simultaneously from two distant sites, the brain and the lumbar spinal cord of a rat. The proposed approach involves the use of MB‐SWIFT to excite two different FOVs using two independent coils and two RF amplifiers. Our data demonstrate the benefits of MB‐SWIFT with dual‐FOV capabilities compared to EPI for CNS fMRI without dedicated shimming approaches. Although we used the MB‐SWIFT pulse sequence for simultaneous fMRI study of brain and spinal cord, the results of this work are generalizable also to other zero or ultra‐short TE MRI approaches. In general, the method could be used for simultaneous imaging of other organs as well.

MB‐SWIFT offers the distinct benefit for imaging with virtually no TE and high bandwidth therefore, inherently minimizing sensitivity to frequency offset variations. This is particularly beneficial for CNS fMRI because it enables circumventing the challenges of dynamic shimming required for large FOVs, and it minimizes sensitivity to both motion and signal dropouts because of susceptibility artifacts, which are prominent in spinal cord imaging. Furthermore, with radial acquisitions, sampling of two FOVs can occur with a time‐shift of a single spoke duration (i.e., within 1 ms from each other), and the center of k‐space critical for detecting fMRI signals is consistently sampled in each spoke for each FOV. Overall, MB‐SWIFT enables virtually simultaneous acquisition of two FOVs. Moreover, in MB‐SWIFT the gradients are “on” during the RF pulse, which allows incremental gradient switching that minimizes gradient‐induced artifacts during electrophysiological recordings and provides a nearly silent acquisition, enhancing subject comfort.^18^ Therefore, future applications of this method could be extended, for example, to MRI of different organs combined with multi‐site electrophysiological recordings or to brain fMRI of different subjects positioned simultaneously in one magnet, that is, social MRI.

In this work, we detected activation patterns in both targets, the brain and spinal cord, in relevant areas in response to electrical hindlimb stimulation. We stimulated the branches of sciatic nerve that originates from spinal cord levels L4 and L5,^32^ where we detected the spinal cord responses, supported also by previous findings.^33^, ^34^ The spinal cord responses detected in this work are similar to those observed in rats by fMRI during hindpaw stimulation^35^ as well as to those detected by functional ultrasound in rodent and swine models during epidural electrical spinal cord stimulation.^36^, ^37^ In particular, the latter reports demonstrate that the hemodynamic responses induced by electrical epidural stimulation in dorsal areas of the spinal cord are faster as compared to the ventral spinal cord. Such an observation was attributed to specific vasculature supplies to the spinal cord with predominantly segmental organization of the ventral part while columnal of the dorsal regions, therefore, leading the anterior and posterior arteries to provide blood supply relatively independently to ventral and dorsal areas of the spinal cord. In humans, the temporal dynamics of the functional signal in the spinal cord manifest a wider and delayed response to tasks as compared to the brain.^38^ All in all, these prior findings are consistent with ours, and suggest that the different vasculature organization, which the MB‐SWIFT technique is particularly sensitive to, may at least partly explain the different fMRI responses between brain and spinal cord. However, further experimental investigations and theoretical modeling of neurovascular coupling phenomena are needed to advance our understanding of the different responses. Such detailed mechanistic studies were outside the scope of the current investigation, which primarily focused on demonstrating the feasibility of the dual‐FOV methodology to conduct simultaneous fMRI studies of brain and spinal cord.

In accordance with previous brain fMRI studies during electrical stimulation of the hindlimb, we observed functional activation in the somatosensory cortex^39^, ^40^, ^41^, ^42^ and in cerebellum.^34^, ^43^ However, we did not observe responses in thalamus, which is part of the sensorimotor network,^44^ activated especially with noxious stimulation.^45^ Lack of thalamic response is relatively common finding in studies of anesthetized animals with non‐noxious sensory stimulus.^41^, ^42^ It is known that the activation of the ventral posterolateral (VPL) nucleus in thalamus is required to activate the primary cortex. However, the detection of the activation in the VPL is challenging because of small dimensions of this section. As such, with BOLD‐fMRI the detection of thalamic activation had been found to be scarce as compared to the other areas of somatosensory pathway, the somatosensory cortex and cerebellum.^46^, ^47^

The ability to detect an fMRI signal simultaneously from distinct sites along the sensory pathway is unique to this technique and could be particularly valuable, for example, for assessing recovery and treatment effects after spinal cord injury or in assessing pain processing. Additionally, when comparing the results obtained with dual‐FOV versus single‐FOV acquisitions, the functional contrast appears very similar, confirming that the quality of the images and the sensitivity to functional activity remains the same when acquiring multiple FOVs. Finally, when compared to the standard SE‐EPI fMRI sequence, the benefit of MB‐SWIFT is in obtaining artifact‐free images with minimal shimming also from two FOVs simultaneously.

### 4.1 Limitations

Several limitations of this study should also be indicated. For shimming, we paid particular attention to minimize the offset frequency for both channels simultaneously, considering that performing the shimming of an extended area covering both FOVs is very challenging. In our set‐up, this choice was appropriate because the MB‐SWIFT pulse sequence tolerates the line broadening if it is smaller than BW/N, where the BW is typically 192 kHz for MB‐SWIFT, and N is the number of voxels along one dimension (frequently equal to 64 for fMRI experiments). Therefore, the half maximum linewidth of the water proton signal must be smaller than 3 kHz, which is a very modest requirement, especially in comparison with EPI type acquisition, which imposes a requirement for linewidths < 50 to 100 Hz.

Additionally, physiological noise and body movement reduce image SNR and fMRI contrast. We used spinal cord supports in our set‐up to limit the effect of the breathing movement in the fMRI spinal cord signal, but further development of the set‐up is needed to improve reproducibility of the spinal cord functional activity. With the current setup, we detected activation in the spinal cord in seven of eight cases with threshold set to tfce z > 3.1. Using a lower threshold, we could see activation also in that animal. The lack of significant activation was likely because of the movement (single larger movement, which was not removed by our motion correction protocol) (Table S2). The current analysis pipeline includes motion correction and removal of components representing motion, however other established approaches to model physiological noise in the fMRI data (e.g., RETROICOR),^48^ could be explored to increase fMRI sensitivity in the spinal cord.

The reconstruction process used for the MB‐SWIFT data results in slightly smooth images. When the number of spokes is reduced to shorten volume‐TR as in the case of fMRI, the radial acquisition may lead to image blurring because of apodization performed during gridding of the undersampled k‐space periphery. In addition, to reduce Gibbs ringing, the high frequency ends of the spokes are scaled down in k‐space. This has the effect of smoothening the image. It should be noted that we made all possible efforts to make sure that the results are not affected, for instance, by co‐registration issues and the brains and spinal cords were well aligned after co‐registration pipeline. However, some responses, for instance, in the cerebellum and in the spinal cord, which were observed in the individual animals were not detected on group level. We attribute this to some differences in the locations of the activation at the individual level, which led them not to be detected with sufficient level of significance at the group level.

Finally, the size of the gradient coils in the Z direction relative to the object should also be considered. The maximal distance between FOVs is limited by the size of the X, Y, and Z gradient coil of the system as well as the overall magnet homogeneity. Notably, if the location of the object is close to the edges of the gradient coil where the gradients start to decline, the images could be distorted with the possibility to create folding artifacts, which could lead to the brightening of the signal. We dedicated particular attention to this aspect when positioning the FOVs. In some cases, the distortion of the image or the presence of one bright artifact can be observed at the edge of the image. However, this artifact did not have any impact on the activation results presented in this work and did not have any concerns for the experimental data.

In this work, with the goal of detecting hindlimb stimulation responses in the brain and spinal cord, we targeted two distant FOVs, namely the rat brain using the first coil, and the lumbar spinal cord with the second coil. Yet the developed method can be adapted to other setups, including different animal models (e.g., mice and swine, and humans) as well as other organs. In these different cases, hardware design and impact of setup on contrast generation need to be carefully taken into account. For instance, in our distant FOVs setup, it was possible to minimize coil coupling using the optimally sized RF shield loop. However, when positioning the two coils closer to each other, the coupling becomes stronger, and the RF shield may not be practical. Other solutions may, therefore, be necessary, such as using active decoupling. An alternative is to prescribe a single large FOV that encompasses both coils, while splitting the RF pulses to two coils. This solution is more time efficient than the current approach, but it works only if the coils are relatively close to each other. Another important consideration should be given to blood saturation effects. With the locations used in this work, the saturation of the blood operated by the coil in the spinal cord was not affecting the signal in the brain. However, when the distance between two coils becomes sufficiently small, and if both coils are placed above or both below the level of the heart, the blood experiencing RF pulses of the first coil may be still saturated when it reaches the FOV of the second coil, thus attenuating the detected responses.

## 5 CONCLUSIONS

We demonstrated the feasibility of dual‐FOV fMRI based on the zero‐TE MB‐SWIFT technique in task‐based fMRI. This methodology opens a new investigational window to study the functional synchronization of brain and spinal cord activity during specific tasks. The method could be used for fMRI investigations of the CNS in basic and clinical neuroscience, including several disorders that manifest in both the brain and spinal cord such as chronic pain, spinal cord injury, and neurodegenerative diseases.

## Conflicts of Interest

Co‐author Javier Istúriz is from Neos Biotec, Pamplona, Spain, which is a company building preclinical RF‐coils. The RF‐coils and the RF‐shield loop used in this study are provided by Neos Biotec.

## Supporting information


**Table S1.** The cluster size and average z‐value of the activated area in the cortex and spinal cord in individual animals.

**Table S2.** Parameters of relative motion in pixels in the spinal cord FOVs in individual animals before and after correcting for motion.

**Figure S1.** The response curves from the brain somatosensory cortex taken from the activated areas from individual animals. The responses from each stimulation block depicted with different colors.
**Figure S2.** The response curves from the spinal cord taken from the activated areas from individual animals. The responses from each stimulation block depicted with different colors. The time courses of rat #4 are not shown because the responses were not significant.

## Data Availability

The data are available at https://doi.org/10.23729/fd‐99ed6084‐2f3e‐37c8‐a4ae‐3cf431be7dd9.
