# Supplementary material for: Simultaneous zero echo time fMRI of rat brain and spinal cord
Source: Magn Reson Med. 2025 Jul 17;94(6):2335–46. doi: 10.1002/mrm.30633 (PMC12283057; doi:10.1002/mrm.30633)

**Supplementary Table 1. The cluster size and average z-value of the activated area in the cortex and spinal cord in individual animals.**


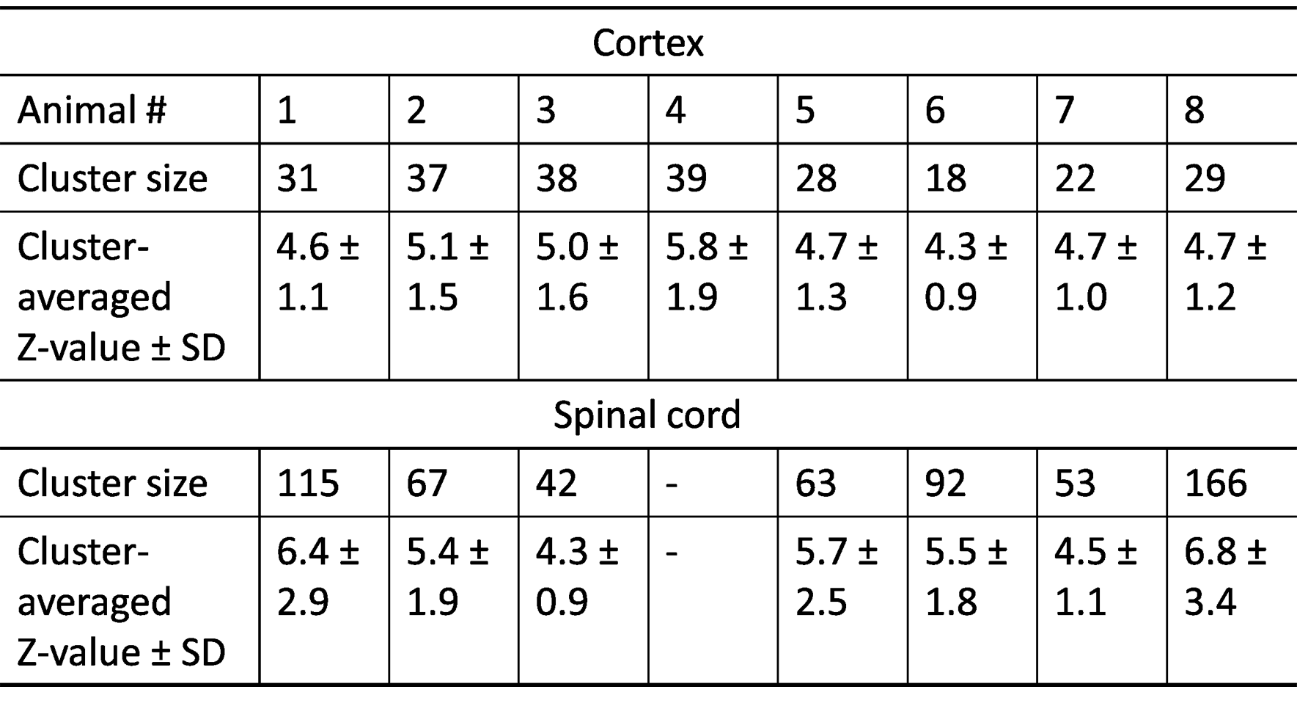

Supplement: Supplementary file 1 — Table S1. The cluster size and average z‐value of the activated area in the cortex and spinal cord in individual animals. [file MRM-94-2335-s003.docx]
