# Supplementary material for: Simultaneous zero echo time fMRI of rat brain and spinal cord
Source: Magn Reson Med. 2025 Jul 17;94(6):2335–46. doi: 10.1002/mrm.30633 (PMC12283057; doi:10.1002/mrm.30633)

**Supplementary Table 2. Parameters of relative motion in pixels in the spinal cord FOVs in individual animals before and after correcting for motion.**


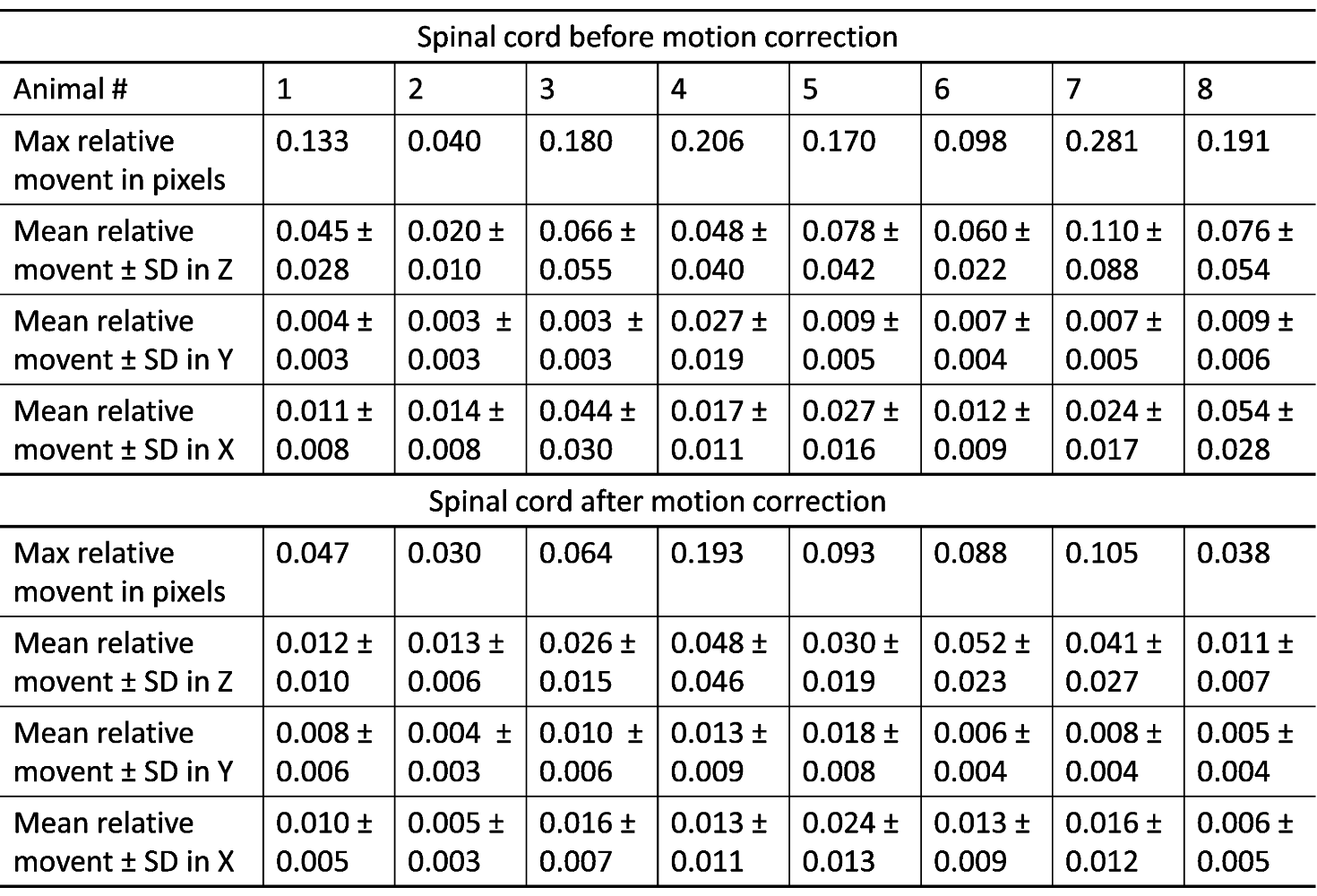

Supplement: Supplementary file 2 — Table S2. Parameters of relative motion in pixels in the spinal cord FOVs in individual animals before and after correcting for motion. [file MRM-94-2335-s001.docx]
