# Supplementary material for: Simultaneous zero echo time fMRI of rat brain and spinal cord
Source: Magn Reson Med. 2025 Jul 17;94(6):2335–46. doi: 10.1002/mrm.30633 (PMC12283057; doi:10.1002/mrm.30633)
Supplement: Supplementary file 3 — Figure S1. The response curves from the brain somatosensory cortex taken from the activated areas from individual animals. The responses from each stimulation block depicted with different colors. Figure S2. The response curves from the spinal cord taken from the activated areas from individual animals. The responses from each stimulation block depicted with different colors. The time courses of rat #4 are not shown because the responses were not significant. [file MRM-94-2335-s002.docx]

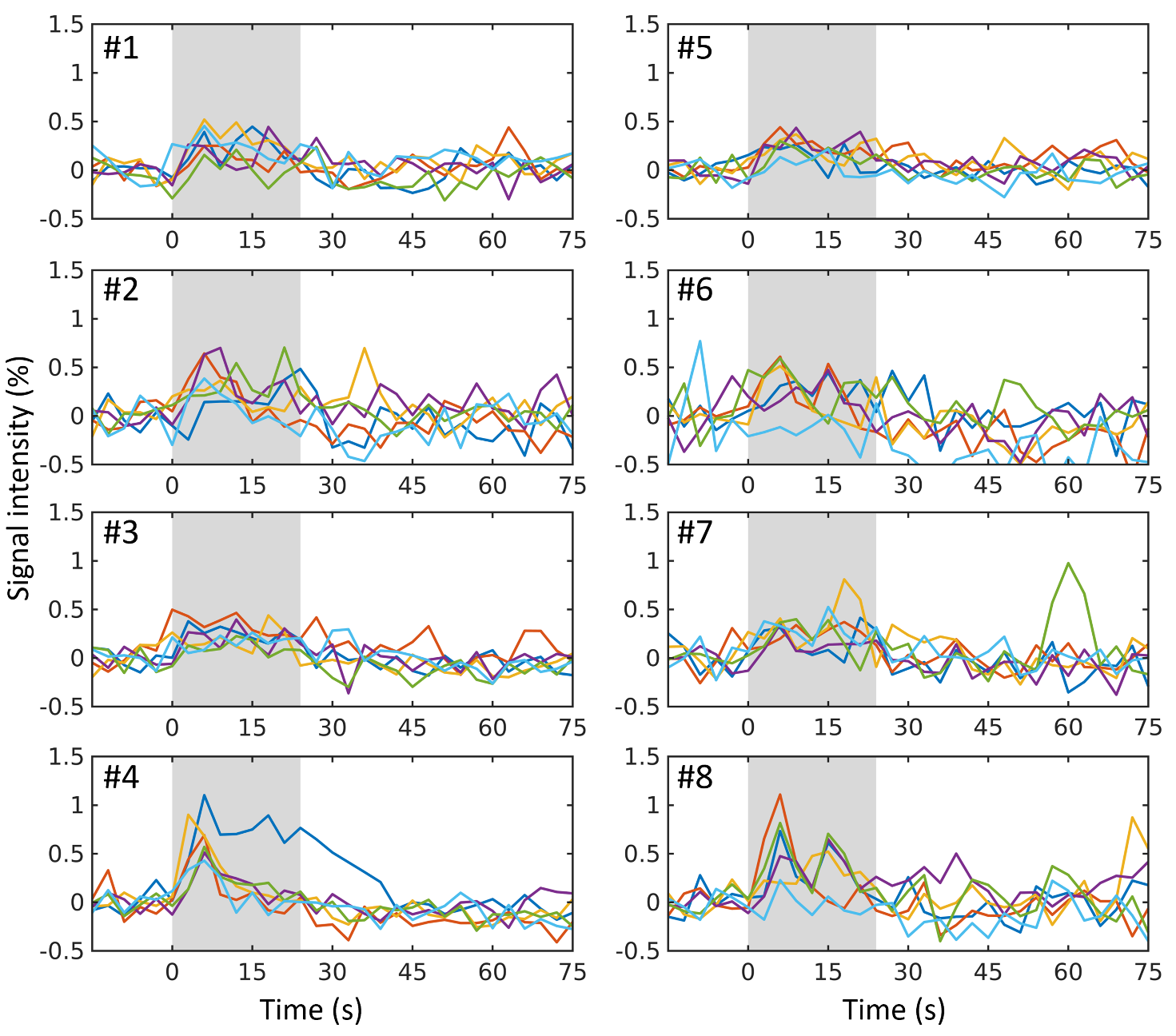
**Figure S1.** **The response curves from the brain somatosensory cortex taken from the activated areas from individual animals.** The responses from each stimulation block depicted with different colors.


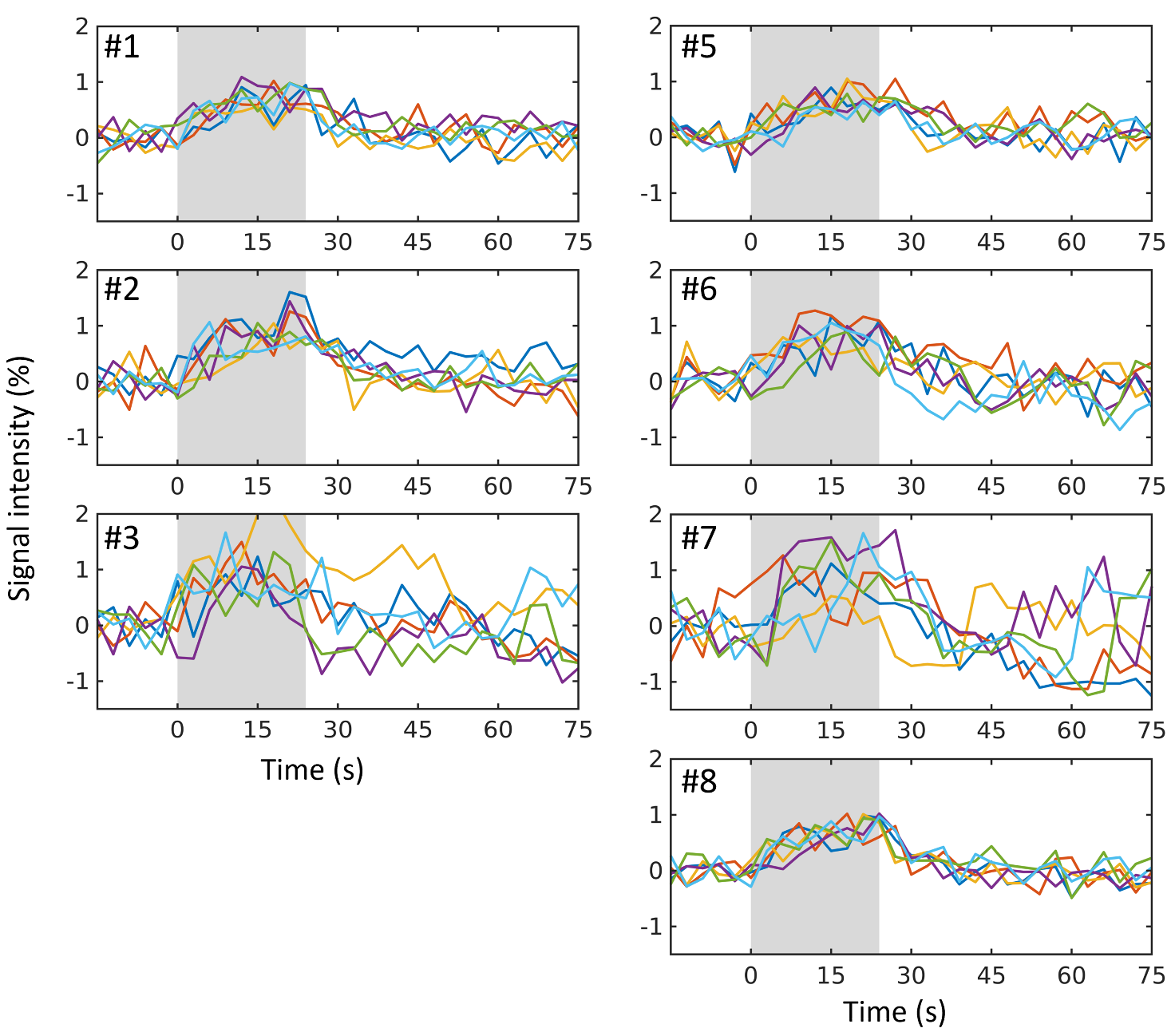
**Figure S2. The response curves from the spinal cord taken from the activated areas from individual animals.** The responses from each stimulation block depicted with different colors. The time courses of rat #4 are not shown because the responses were not significant.
